# Phylogenetic and Pathogenic Analyses of Avian Influenza A H5N1 Viruses Isolated from Poultry in Vietnam

**DOI:** 10.1371/journal.pone.0050959

**Published:** 2012-11-30

**Authors:** Dongming Zhao, Libin Liang, Yanbing Li, Yongping Jiang, Liling Liu, Hualan Chen

**Affiliations:** State Key Laboratory of Veterinary Biotechnology, Harbin Veterinary Research Institute, Chinese Academy of Agricultural Sciences, Harbin, People’s Republic of China; Duke-NUS Graduate Medical School, Singapore

## Abstract

Despite great efforts to control the infection of poultry with H5N1 viruses, these pathogens continue to evolve and spread in nature, threatening public health. Elucidating the characteristics of H5N1 avian influenza virus will benefit disease control and pandemic preparation. Here, we sequenced the genomes of 15 H5N1 avian influenza viruses isolated in Vietnam in 2006 and 2007 and performed phylogenetic analyses to compare these sequences with those of other viruses available in the public databases. Molecular characterization of the H5N1 viruses revealed that seven genetically distinct clades of H5N1 viruses have appeared in Vietnam. Clade 2.3.4 viruses existed in Vietnam as early as 2005. Fifteen viruses isolated during 2006 and 2007 belonged to clade 1 and clade 2.3.4, and were divided into five genotypes. Reassortants between the clade 1 and clade 2.3.4 viruses were detected in both North and South Vietnam. We also assessed the replication and pathogenicity of these viruses in mice and found that these isolates replicated efficiently and exhibited distinct virulence in mice. Our results provide important information regarding the diversity of H5N1 viruses in nature.

## Introduction

Highly pathogenic avian influenza (HPAI) H5N1 viruses have now spread through poultry populations in many countries. These viruses have also crossed species barriers to infect different hosts [1]–[4]. HPAI H5N1 viruses have repeatedly shown their potential to be transmitted directly from birds to humans [5] and still pose a significant threat to human health. In retrospect, most patients infected by HPAI H5N1 viruses had direct or indirect exposure to sick or dead poultry (WHO [http://www.who.int]). Influenza A virus continuously mutates while circulating in nature and overcomes host immunity from previous infections, posing great challenges to disease control [6]–[11].

Vietnam is one of the highest frequencies of HPAI H5N1 outbreaks. HPAI H5N1 virus was first identified in Vietnam in 2001 [12], and outbreaks in poultry have been reported in more than 59 of the 64 Vietnamese provinces since December of 2003 (OIE, 2010). The first human infection in Vietnam was reported in 2004; by August of 2012, 123 cases and 61 deaths had been reported (WHO [http://www.who.int]). Nationwide vaccination programs and culling strategies have been performed to control the disease, which has greatly contributed to a reduction in outbreaks. But despite these great efforts to control the disease, HPAI H5N1 viruses continue to evolve and cause outbreaks in poultry and human infections in Vietnam.

To better understand the molecular and biological properties of H5N1 avian influenza viruses, we selected 15 H5N1 strains isolated from poultry in Vietnam during 2006 and 2007 and sequenced their entire genomes. We performed phylogenetic analyses combining with the sequence data from the Vietnam influenza viruses and other representative viruses available in public databases, and then genotyped the viruses on the basis of their whole genomes. We also assessed the replication and pathogenicity of these viruses in mice. Understanding the molecular and biological features of avian H5N1 viruses will help reveal the potential evolutionary and transmission features of H5N1 viruses, and benefit disease control and pandemic preparedness.

## Materials and Methods

### Viruses

The 15 HPAI H5N1 viruses used in this study were isolated from domestic poultry, including chickens, Muscovy ducks, and ducks on farms in Vietnam. Details of these viruses are given in Table 1. Virus stocks were propagated and purified in the allantoic cavities of 10-day-old embryonated eggs as previously described [11]. Virus stocks were aliquoted and stored at −80°C until use. All experimental work with H5N1 viruses, including animal studies, was performed in a bio-safety level-3 laboratory approved for such use by the Ministry of Agriculture of China.

**Table 1:** Influenza virus isolates from poultry in Vietnam, 2006–2007.

Isolates*	Province	Position†	Year	Sublineage ‡	M2 ionchannel		
MDK/VN/1185/06	Ca Mau	S	2006	Clade 1	I26		N31
CK/VN/1180/06	Ca Mau	S	2006	Clade 1	I26		N31
MDK/VN/1159/06	Ca Mau	S	2006	Clade 1	I26		N31
MDK/VN/1181/06	Ca Mau	S	2006	Clade 1	I26		N31
DK/VN/1213/07	Bac Lieu	S	2007	Clade 1	I26		N31
CK/VN/1214/07	Bac Lieu	S	2007	Clade 1	I26		N31
CK/VN/20/07	Cao Bang	N	2007	Clade 2.3.4			
MDK/VN/22/07	Ca Mau	S	2007	Clade 2.3.4			N31
DK/VN/31/07	Soc Trang	S	2007	Clade 2.3.4			
DK/VN/34/07	Son La	N	2007	Clade 2.3.4			N31
CK/VN/41/07	Hai Duong	N	2007	Clade 2.3.4			
DK/VN/43/07	Cao Bang	N	2007	Clade 2.3.4	I26	A27	N31
CK/VN/44/07	Ha Tay	N	2007	Clade 2.3.4			
CK/VN/45/07	Hanoi	N	2007	Clade 2.3.4			
MDK/VN/46/07	Hai Duong	N	2007	Clade 2.3.4			

* MDK, Muscovy duck; CK, chicken; DK, duck; VN, Vietnam.

† The letters S and N denote southern Vietnam and northern Vietnam, respectively.

‡ Based on the World Health Organization influenza (H5N1) nomenclature system.

### Genomic Sequencing and Phylogenetic Analysis

Viral RNA was extracted from allantoic fluid by using TRIZOL Reagent (Invitrogen), and was then reverse-transcribed. A set of fragment-specific primers (primer sequences available on request) was used for the PCR amplification and sequence analysis. The PCR products were purified with the Watson PCR purification kit (Watson) and sequenced by using the CEQ DTCS-Quick Start Kit on a CEQ 8800 DNA sequencer (Beckman Coulter). Sequence data were compiled with the SEQMAN program (DNASTAR, Madison, WI). Phylogenetic trees were generated with MEGA 4.0 by neighbor-joining (NJ) methods and bootstrap tests (1000 replicates; seed = 64238) based on the sequences for the open reading frames (ORFs).

### Nucleotide Sequence Accession Numbers

The nucleotide sequences obtained in this study are available from GenBank (accession nos. JX420123- JX420242).

### Infection of Mice

Groups of eight six-week-old female BALB/c mice (Beijing Experimental Animal Center, Beijing) were lightly anesthetized with CO_2_ and inoculated intranasally with 10^6.0^ EID_50_ of H5N1 influenza virus in a volume of 50 µL [11]. Control mice were inoculated with PBS. On day 3 post-inoculation (p.i.), three of the eight mice in each group were euthanized and their organs, including lung, kidney, spleen, and brain, were collected and homogenized in 1 mL of cold PBS by using a Tissue Lyser (QIAGEN). Solid debris was pelleted by centrifugation, and undiluted and 10-fold serially diluted supernatants were inoculated in 10-day-old embryonated eggs. The titers for virus infectivity in eggs were calculated by the method of Reed and Muench [13], [14]. The remaining five mice in each group were monitored daily for weight loss and mortality for 14 days. The 50% mouse lethal dose (MLD50) was determined by inoculating groups of five mice with 10-fold serial dilutions containing 10^1.0^ to 10^6.0^ EID50 of virus in a volume of 50 µL and calculated by using the method of Reed and Muench [13], [14].

### Ethics Statements

This study was carried out in strict accordance with the recommendations in the Guide for the Care and Use of Laboratory Animals of the Ministry of Science and Technology of the People’s Republic of China. The protocol was approved by the Committee on the Ethics of Animal Experiments of the Harbin Veterinary Research Institute, Chinese Academy of Agricultural Sciences (approval number: BRDW-XBS–02).

## Results

### Molecular and Phylogenetic Analysis

**Figure 1:**
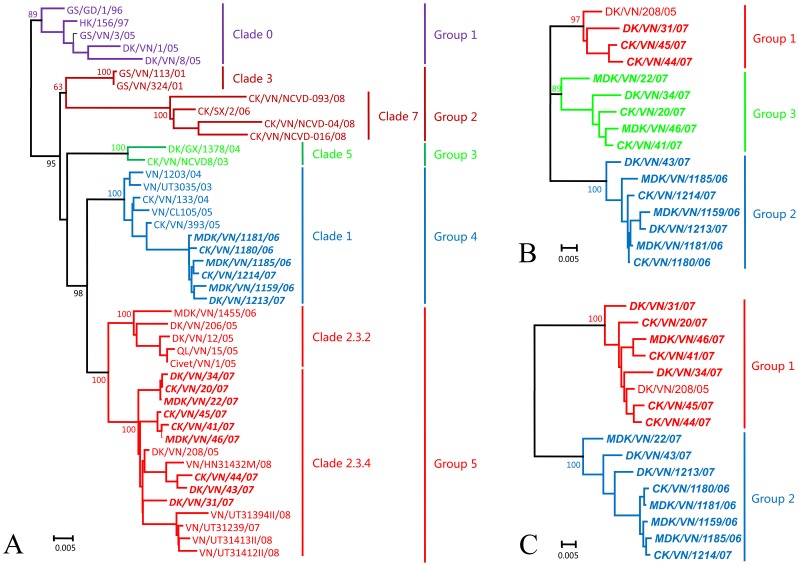
Phylogenetic trees for the HA (A), NA (B), and PA (C) genes of the H5N1 influenza A viruses analyzed. The trees were generated by using CLUSTALx1.83 and MEGA4.0 software by the NJ method (Bootstrap test:1000 replicates, seed = 64238 ) on the basis of the following gene sequences: nucleotides 29–1,695 (1,667 bp) of HA, 21–1,358 (1,338 bp) of NA, and 25–2,163 (2,139 bp) of PA. The length of each pair of branches represents the distance between the sequence pairs, and the units at the bottom of the tree indicate the number of substitution events. The 15 H5N1 isolates from Vietnam are marked in bold italic. Abbreviations: CK, chicken; DK, duck; MDK, Muscovy duck; QL, quail; GS, goose; GD, Guangdong; GX, Guangxi; HK, Hong Kong; VN, Vietnam; SX, Shanxi.

To determine the molecular features of H5N1 avian influenza viruses in Vietnam, all eight gene segments of the 15 viruses were sequenced and those sequences were compared with the sequences in public databases of 25 representative influenza viruses from Vietnam and 4 representative viruses from China. The HA genes of these 44 viruses belonged to seven different clades of the WHO influenza (H5N1) nomenclature system (WHO, 2010), which could be further divided into five different groups on the basis of their evolutionary relationships (Figure 1A).

Group 1 contained five clade 0 viruses, three of which were isolated from the eggs of Vietnamese waterfowl in 2005 [15]. Group 2 comprised two clade 3 viruses, GS/VN/113/01 and GS/VN/324/01, which were detected in 2001 in Vietnam, and four clade 7 viruses, three of which were detected in Vietnam in 2008 [16], [17], and another one was isolated from China. Group 3 contained two clade 5 viruses that were isolated in Vietnam in 2003 and 2004, respectively. Group 4 contained 11 clade 1 viruses that were isolated from humans and poultry in Vietnam during 2003–2007. Group 5 contained five clade 2.3.2 viruses and 15 clade 2.3.4 viruses. Four of the five clade 2.3.2 viruses were isolated in 2005 and one was isolated in 2006. The isolation of clade 2.3.2 viruses from domestic poultry and civet indicated that these viruses existed widely in Vietnam as early as 2005. Of the 15 clade 2.3.4 viruses, 14 were isolated from domestic poultry and humans in 2007 and 2008, whereas one strain, DK/VN/208/05, was detected in ducks in Vietnam as early as 2005. The HA gene of six of the 15 viruses we sequenced in this study belonged to clade 1, whereas the HA gene of the other nine viruses belonged to clade 2.3.4 (Fig 1A).

Analysis of the deduced amino acid sequences of the HA genes showed that all of the 15 isolates sequenced had a series of basic amino acids at the HA cleavage site (-RRRKR/−RRKKR-), a characteristic of highly pathogenic influenza viruses in chickens [18]. Most isolates had seven sites of potential glycosylation, five sites in HA1 and two in HA2, based on our analysis of the deduced amino acid sequences [19], [20]; however, CK/VN/1214/07 did not have a glycosylation site at 170, and DK/VN/1213/07 did not have a glycosylation site at 559. The amino acids at positions 226 and 228 of the HA of all of the viruses were Gln and Gly, respectively, implying that these viruses may retain the characteristic of preferentially binding to avian-like α2, 3-NeuAcGal linkages [21], [22].

The NA genes of the 15 strains sequenced and DK/VN/208/05 mainly formed three groups (Figure 1B). Homology among the genes within these groups was over 97%, but the percent identity of the genes was less than 97% among the different groups. Group 1 contained four viruses, DK/VN/208/05, CK/VN/44/07, CK/VN/45/07, and DK/VN/31/07. Seven of the 15 strains sequenced formed group 2, six of which were isolated in Southern Vietnam. However, DK/VN/43/07, which was isolated in Northern Vietnam, also belonged to group 2. The NA genes of the other five viruses were in group 3. Analysis of the deduced amino acid sequences of the NA genes demonstrated that all 15 viruses had a 20-amino acid deletion (residues 49–68) in the stalk of their NA proteins. Mutations in NA associated with oseltamivir or zanamivir resistance were not detected [23], [24].

The PA genes of the 15 sequenced strains mainly formed two groups (Figure 1C). Group 1 contained six viruses isolated from Northern Vietnam and DK/VN/31/07 from the South. Group 2 contained seven viruses isolated from Southern Vietnam and DK/VN/43/07 from the North. Homology among the genes within these groups was over 97%, but the homology of the genes between the two groups was less than 95%.

The PB2 and PB1 genes of the 15 strains also formed two groups, similar to those of the PA genes; however, the PB2 and PB1 genes of MDK/VN/22/07 were included in group 1. Similarly, the same trends were seen in the phylogenetic tree for the NS genes of the viruses. The NP and M genes of all of these viruses shared over 97% homology and were considered to belong to one lineage. Mutations at certain positions in the M2 protein, including I26, A27, and N31, were associated with the amantadine resistance of influenza viruses [25], [26]. The I26 and N31 mutations were detected in the M2 protein of all six of the clade 1 viruses and in one clade 2.3.4 virus (Table 1); the N31 mutation was also detected in the M2 protein of two clade 2.3.4 viruses (Table 1). It is interesting to note that, in addition to the I26 and N31 mutations in the M2 protein, the DK/VN/43/07, a clade 2.3.4 virus, also contained the A27 mutation. The deduced NS amino acid sequences of the 15 strains had a 15-nucleotide deletion that resulted in a 5-amino acid deletion in the NS protein (amino acid positions 80–84) [11].

**Figure 2:**
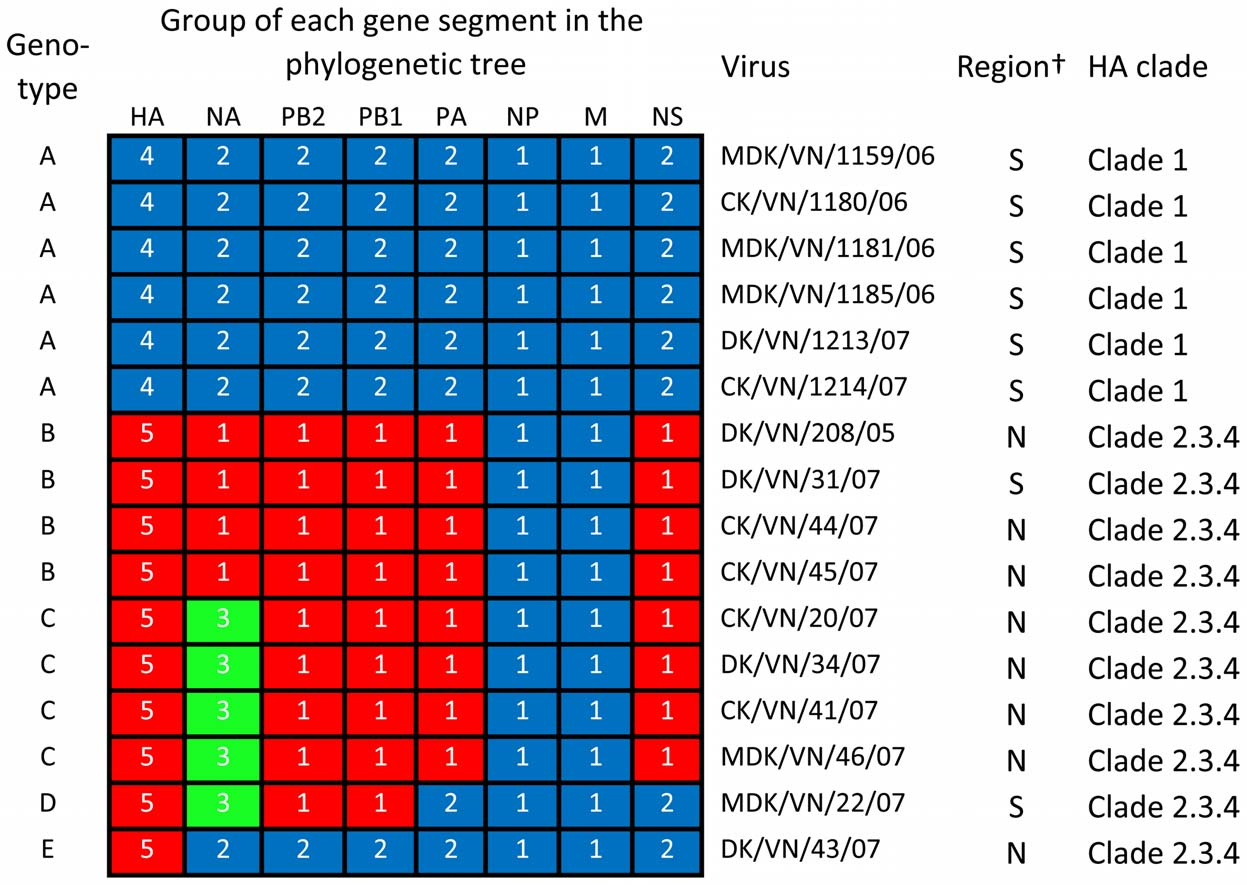
Genotypic evolution of H5N1 viruses isolated from poultry in Vietnam in 2006 and 2007. The eight gene segments are indicated at the top of each bar. The number in each bar shows the group of genes indicated in Figure 1. DK/VN208/05 was used in this analysis because it represents the earliest clade 2.3.4 isolate in Vietnam in the public databases to date. † The letters S and N denote southern Vietnam and northern Vietnam, respectively.

On the basis of genomic diversity, the 15 strains sequenced in this study were divided into five genotypes (Fig. 2). Genotype A contained six clade 1 viruses. Genotype B contained the three clade 2.3.4 viruses analyzed in this study and DK/VN/208/05, which was reported previously by others [27]. Genotype C contained four viruses, which were reassortants of genotype B viruses that contributed new NA genes. Genotype D contained one virus, which was a reassortant virus of a genotype C virus that derived its PA and NS genes from a genotype A (clade 1) virus. Genotype E contained one virus that had the HA gene of a clade 2.3.4 virus and its remaining seven genes from a genotype A (clade 1) virus. These results indicated that reassortment between clade 1 and calde 2.3.4 viruses actively occurred in Vietnam.

### Studies with Mice

**Figure 3:**
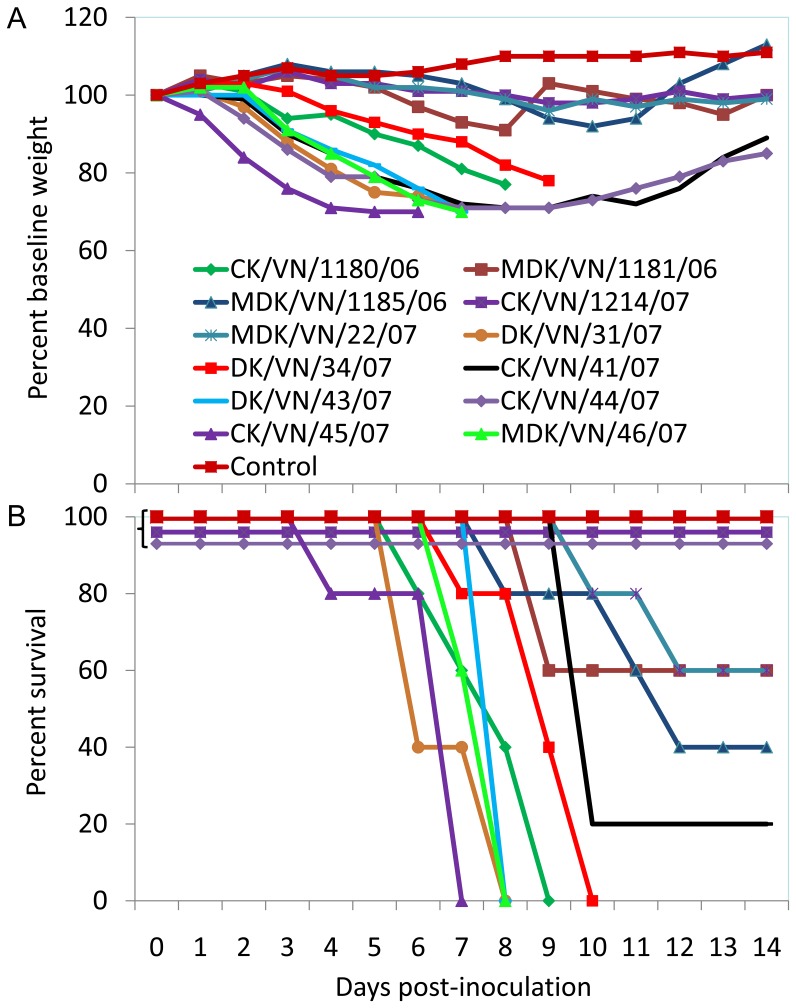
Replication and virulence of H5N1 influenza viruses in mice. (A) Weight changes of mice inoculated with different H5N1 viruses. Groups of five mice were intranasally inoculated with 10^6^ EID_50_ (50 µL) or with PBS as a control and weighed daily for 14 days. (B) Survival percentage of mice inoculated with H5N1 viruses.

To determine the ability of H5N1 avian influenza viruses to replicate and be pathogenic in mammals, we tested 12 viruses in mice. Groups of 8 mice were inoculated intranasally with 10^6^EID_50_ of virus; three mice were euthanized on day 3 p.i. and their organs, including lung, spleen, kidney, and brain, were collected for virus titration in eggs, while the remaining five mice were observed for two weeks for changes in body weight or signs of death. As shown in Table 2, six viruses, including CK/VN/1180/06, MDK/VN/1181/06, DK/VN/31/07, DK/VN/43/07, CK/VN/45/07, and MDK/VN/46/07, were detected in the brains, spleens, kidneys and lungs of mice, and some mice showed severe neurological dysfunction. One virus, DK/VN/34/07, was detected in the spleens, kidneys and lungs but not brains of mice. Four strains, MDK/VN/1185/06, MDK/VN/22/07, CK/VN/41/07, and CK/VN/44/07, were detected in the lungs and spleens of mice, and CK/VN/1214/07 was only detected in the lungs of mice. Mice inoculated with six viruses, including CK/VN/1180/06, DK/VN/31/07, DK/VN/34/07, DK/VN/43/07, CK/VN/45/07, and MDK/VN/46/07, lost over 20% of their body weight and died within 10 days of infection (Figure 3 A, B, and Table 2). Mice inoculated with CK/VN/41/07 and CK/VN/44/07 also experienced over 20% body weight loss; CK/VN/41/07 killed three of the five mice, whereas CK/VN/44/07 did not kill any during the two-week observation period (Figure 3 A, B, and Table 2). Mice inoculated with the four viruses MDK/VN/1185/06, MDK/VN/1181/06, MDK/VN/22/07, and CK/VN/1214/07 lost less than 10% of their body weight, and 2–4 of the five mice died in the MDK/VN/1181/06-, MDK/VN/1185/06-, and MDK/VN/22/07- inoculated groups. None of the mice that were infected with the CK/VN/1214/07 virus died during the observation period (Figure 3 A, B, Table 2). We further selected five of the viruses that killed all of the mice after inoculation with the dose of 10^6^EID_50_, and determined their 50% mouse lethal doses (MLD_50_) as described previously [13]. The MLD_50_ values for these five viruses ranged from 2.5–3.5log_10_EID_50_ (Table 2). These results indicated that the viruses circulating in the poultry in Vietnam are able to replicate in mice, and some of them are highly lethal for mice. .

**Table 2:** Replication and virulence of H5N1 viruses in mice.^a.^

Virus	Genotype	Virus replication in organs (log_10_EID_50_/mL ± SD^b^)				No. of deadmice^c^	MLD_50_
		Lung	Spleen	Kidney	Brain		
MDK/VN/1185/06	A	4.6±0.3 d	+^e^	−	−	3	−f
CK/VN/1180/06	A	5.6±0.1	2.8±0.8	2.8±0	2.7±0.0	5	2.5
MDK/VN/1181/06	A	4.8±0.7	+	+	+	3	−
CK/VN/1214/07	A	5.0±0.2	−	−	−	0	>6.5
MDK/VN/22/07	D	4.8±0.7	+	−	−	2	6.2
DK/VN/31/07	B	6.6±0.1	4.2±0.7	3.8±0.2	2.3±0.5	5	2.6
DK/VN/34/07	C	5.8±0.3	3.8±0.5	1.4±0.0	−	5	−
CK/VN/41/07	C	6.2±0.6	2.3±0.9	−	−	4	−
DK/VN/43/07	E	5.7±0.9	+	+	+	5	3.5
CK/VN/44/07	B	5.6±0.1	1.8±0.4	−	−	0	>6.5
CK/VN/45/07	B	6.4±0.1	2.7±0.0	2.2±0.7	1.7±0.1	5	2.6
MDK/VN/46/07	C	6.9±0.5	3.4±0.1	2.8±0.6	2.6±0.8	5	3.2

a Six-week-old BALB/c mice were used for this study.

b Standard deviation.

c The data were acquired when mice were inoculated intranasally with 10^6^ EID_50_ of H5N1 virus in a volume of 50 µL.

d The titer shown are the means ± standard deviations of the mice inoculated.

e +, Viruses were only detected from undiluted samples; -, the viruses were not detected in the organs.

f The data were not acquired.

## Discussion

HPAI H5N1 viruses were detected in Vietnam as early as 2001 [12]. Our study here indicates that multiple clades of H5N1 viruses, including clade 0, clade 1, clade 2.3.2, clade 2.3.4, clade 3, clade 5, and clade 7, have been detected or are still circulating in poultry in Vietnam. Outbreaks in poultry and human infections detected in Vietnam have mainly been caused by clade 1 and clade 2.3.4 viruses [28]. The clade 2.3.4 viruses were detected in Vietnam as early as 2005, which disagrees with a previous report that the clade 2.3.4 viruses were first detected in Vietnam in 2007 (Wan et al, 2008). The clade 0, calde 3, clade 5, and clade 7 viruses have been detected in poultry or poultry products in Vietnam [17], [29], however, it seems that these strains circulated silently in poultry and were not related to any reported disease outbreaks. From the phylogenetic tree of the HA genes, it appears that the clade 3 and clade 7 viruses are closely related, and the clade 7 viruses may have evolved from the clade 3 viruses or that these two viruses may share a common ancestor.

Analysis of the 15 viruses sequenced in this study further revealed that the dominant viruses circulating in Vietnam in 2006 and 2007 belonged to clade 1 and clade 2.3.4. A previous study reported that HPAI H5N1 viruses were concentrated in specific geographical regions, with clade 1 viruses mainly in Southern Vietnam and clade 2.3.4 viruses mainly in Northern Vietnam [30]. However, in our study we found that some clade 2.3.4 viruses also appeared in Southern Vietnam, such as MDK/VN/22/07 and DK/VN/31/07. The fact that at least five genotypes of H5N1 viruses bearing gene segments of clade 1 and clade 2.3.4 viruses or the NA gene of unknown viruses were circulating in poultry (mainly ducks) suggests that multiple subtypes of influenza viruses may have actively co-circulated in waterfowl in Vietnam and that reassortment among different viruses occurred frequently.

Most Eurasian HPAI viruses isolated since 1997 can replicate in mammals [31], [32]. In previous studies, we observed increased pathogenicity among H5N1 viruses isolated from ducks when tested in mice [11], and Maines et al. (2005) reported that HPAI H5N1 viruses display increased virulence in mammals [33]. The pathogenicity analysis in this study showed that the 12 viruses tested could replicate efficiently in mice without prior adaptation and exhibited different pathogenic potential in mice. In addition, we found no direct relationship between viral genotype and pathogenicity in mice.

The virulence of influenza virus is determined by multiple gene products and amino acid sites. Several determinant sites in the PB2, PA, HA, NS1, and M1 genes are associated with the virulence of avian influenza viruses in mammals [13], [34]–[37]. All of the 15 viruses we characterized have the same amino acids at positions that are known to be crucial for the virulence of H5N1 influenza virus in mice; however, the virulence of CK/VN/1214/07 and of CK/VN/44/07 was more than 1,000-fold lower in mice than that of the other viruses of the same genotype (Figure 3 and Table 2). Therefore, other unknown determinants of virulence appear to be involved in the high pathogenicity of these viruses in mice. These viruses could, therefore, serve as models to explore new factors responsible for the high virulence of H5N1 viruses in mammals.

In summary, we characterized the genetic and biological diversity of HPAI H5N1 viruses isolated in Vietnam and uncovered important information that contributes to our comprehensive understanding of these viruses. Influenza viruses spread in nature all year around in tropical regions [38], [39]. The multiple clades and genotypes of the viruses that have appeared in Vietnam suggest that environmental factors, such as high humidity, heavy poultry population, undeveloped breeding style, less bio-security in poultry farms, close contact between poultry and other mammalian hosts, in these areas may have facilitated the generation of reassortants and mutants. Accordingly, these HPAI H5N1 viruses may have more opportunities to acquire the ability to efficiently transmit to humans. Clearly, it is of great importance to continuously monitor poultry and to regularly update control strategies.
